# Ebola Virus Disease Complicated by Late-Onset Encephalitis and Polyarthritis, Sierra Leone

**DOI:** 10.3201/eid2201.151212

**Published:** 2016-01

**Authors:** Patrick Howlett, Colin Brown, Trina Helderman, Tim Brooks, Durodamil Lisk, Gibrilla Deen, Marylou Solbrig, Marta Lado

**Affiliations:** Kings Sierra Leone Partnership, Freetown, Sierra Leone (P. Howlett, M. Lado);; University College London Hospital, London, UK (C. Brown);; Medair, Ecublens, Switzerland (T. Helderman);; Public Health England, Porton Down, UK (T. Brooks);; Connaught Hospital, Freetown (D. Lisk, G. Deen);; University of Kansas, Lawrence, Kansas, USA (M. Solbrig)

**Keywords:** Ebola virus disease, Ebola virus, encephalitis, polyarthritis, viral persistence, Sierra Leone, viruses

**To the Editor:** Ebola virus (EBOV) disease is usually an acute illness, but increasing evidence exists of persistent infections and post-Ebola syndromes. We report a case of EBOV encephalitis.

A 30-year-old woman with no known EBOV contact sought treatment at an Ebola isolation unit in Freetown, Sierra Leone, on January 1, 2015 (day 7 of illness). She was afebrile and weak, but ambulatory, with a history of fever, vomiting, diarrhea, headache, and muscle and joint pain. According to local protocol, she was given oral antimalarial, antimicrobial, and antiemetic drugs and oral rehydration therapy. On day 8 of illness, after testing EBOV PCR–positive (cycle threshold [C_t_] value of 23.5) (*1*), she was given intravenous ceftriaxone (2 g) for 7 days, artesunate (180 mg) for 3 days, and Ringer’s lactate (4–6 L) with supplemental KCl for 5 days.

During days 13–15, the patient improved, moving independently and talking. On day 16, she became confused; by day 20, she was unresponsive to voices. Intravenous ceftriaxone (2 g) and artesunate (180 mg) were administered for an additional 7 and 3 days, respectively. On days 28 and 29, she was still unconscious; serum PCR test results on both days were negative for EBOV. On day 29, she was transferred to Connaught Hospital in Freetown, where she had a Glasgow Coma Scale score of 9/15 (E3, V1, M5) but no localizing or focal signs. She was given intravenous fluconazole (800 mg 1×/d). Admission blood test results showed anemia, elevated **alanine aminotransferase** and C-reactive protein, and low creatinine (Technical Appendix). HIV test results were negative. 

On day 34, large-joint polyarthritis of the right shoulder, left elbow, and left knee developed. Affected joints appeared normal on radiographs, and synovial fluid (15 mL) from the left knee was EBOV PCR negative. She was given diclofenac (50 mg 2×/d) and 1 intramuscular dose of methylprednisolone (80 mg). Concurrent blood PCR on day 34 was negative.

By day 41 she was more alert, although her family reported she had slowed responses. Lumbar puncture was performed; opening pressure (30 cm H_2_O) was elevated, and cerebrospinal fluid (CSF) was EBOV PCR–positive (C_t_ value 37.6), as determined by using the Public Health England in-house, optimized version of the Trombley assay (*2*) with a cutoff C_t_ value of 40. Concurrent catheter specimens of urine and blood samples tested EBOV-negative. FilmArray (BioFire Diagnostics, Salt Lake City, UT, USA) testing showed methicillin-resistant *Staphylococcus aureus* and *Klebsiella pneumoniae* in CSF and mixed pathogens in urine. A computer tomographic scan image of the patient’s head showed substantial cerebral atrophy without hydrocephalus (Figure).

**Figure:**
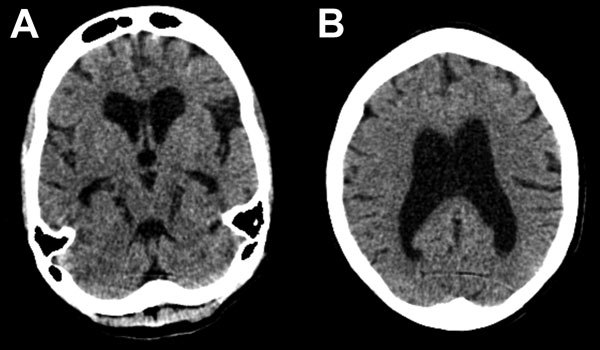
Representative axial cuts from noncontrast head computed tomography scan imaging of a 30-year-old woman with encephalitis resulting from Ebola virus infection, Sierra Leone. Images show global atrophy in keeping with nonobstructive ventriculomegaly and no periventricular low attenuation: A) subcortical atrophy; B) cortical atrophy. There was no evidence of hydrocephalus, previous stroke, or intracranial hemorrhage. A cavum septum pellucidum was noted in other images.

On day 44, an underarm sweat swab sample was PCR-positive (C_t_ value 39.6) and a buccal swab sample PCR-negative for EBOV. Ongoing painful synovitis was treated with an additional 80-mg intramuscular dose of methylprednisolone. On day 51, a midstream urine sample was EBOV PCR–positive (C_t_ value 35.7), and an underarm sweat swab sample was EBOV PCR–negative. The patient was discharged; her family was advised to minimize contact with her body fluids.

At follow-up on day 64, the patient’s family reported she had impaired short-term memory and ongoing slowness. She had a score of 18/23 on the Mini–Mental State Examination, but general neurologic exam results were normal. A midstream urine test was still EBOV PCR–positive (C_t_ value 39.6); PCR of her sweat swab sample was inhibited (Technical Appendix). She was referred to the local survivors’ clinic; no contact cases were reported.

The depressed mental status and presence of EBOV in this case-patient’s CSF are consistent with encephalitis, a finding in autopsies of persons with Marburg virus infection (*3*,*4*) and in EBOV nonhuman primate models (*5*). The general atrophy seen in computer tomographic scan images is consistent with a rapidly developing complication of a diffuse inflammatory process. Given inadequate antimicrobial drug doses for meningitis and clinical improvement, we believe methicillin-resistant *S. aureus* and *K. pneumoniae* were CSF sample contaminants.

This case shows the brain’s immune privilege is incomplete for EBOV and prompts a broader discussion regarding neurovirulence in Ebola virus disease. Our finding that EBOV can be present in CSF, even after serum clearance, adds to the knowledge of neurologic symptoms in acute infection and of postinfectious sequelae in observational clinical studies (*6*–*8*). This finding raises the possibility that EBOV persistence elsewhere in the body, or in multiple organs, could be an indicator of or risk for central nervous system invasion. 

Our report has limitations. We could not perform many blood chemistry tests, in-country virus cultures, or deep sequencing on samples. Likewise, diagnosis of coma was challenging because of the lack of CSF cell counts, biochemistry values, and paired EBOV IgG and IgM titers in CSF and blood.

This case raises the practical issue that Ebola treatment requires understanding of multiorgan virologic and inflammatory complications; survivor care and research programs should screen for neurocognitive impairment and consider appropriate imaging. The case confirms previously reported intermittent EBOV PCR positivity in urine (*9*). The development of arthritis with synovitis, treated with corticosteroids, supports the diagnosis of reactive arthritis.

Technical Appendix Blood test and cycle threshold results for a 30-year-old woman with Ebola virus disease, Sierra Leone.
