# Supplementary material for: Ebola Virus Disease Complicated by Late-Onset Encephalitis and Polyarthritis, Sierra Leone
Source: Emerg Infect Dis. 2016 Jan;22(1):150–2. doi: 10.3201/eid2201.151212 (PMC4696703; doi:10.3201/eid2201.151212)
Supplement: Technical Appendix — Blood test and cycle threshold results for a 30-year-old woman with Ebola virus disease, Sierra Leone. [file 15-1212-Techapp-s1.pdf]

Publisher: CDC; Journal: Emerging Infectious Diseases
Article Type: Letter; Volume: 22; Issue: 1; Year: 2016; Article ID: 15-1212
DOI: 10.3201/eid2201.151212; TOC Head: Letters to the Editor
Article DOI: http://dx.doi.org/10.3201/eid2201.151212

Ebola Virus Disease Complicated by Late Onset Encephalitis and Polyarthritis

Technical Appendix

Technical Appendix Table. Blood test results at admission for a 30-year-old woman with Ebola virus disease, Sierra Leone*

Parameter	Value or result	Reference range or value
Hemoglobin, g/dL	8.4	11.5–16.0
Platelets, x 10^9/L	254	150–400
Leukocyte count, x 10^9/L	5.4	4.0–11.0
Creatinine, µmol/L	34	70–150
Alanine aminotransferase, IU/L	90	5–35
C-reactive protein, mg/L	30	<5
HIV	Negative	NA

*Blood tests were not repeated after admission. NA, not applicable.

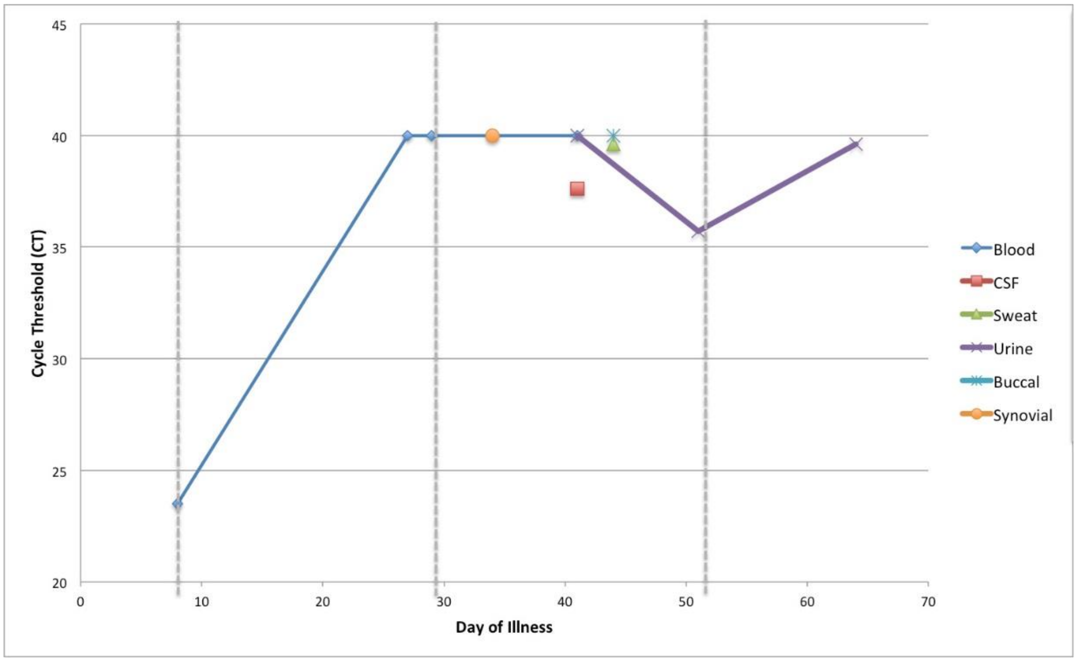

Technical Appendix Figure. Cycle threshold results for Ebola virus in body fluids of a 30-year-old woman with Ebola virus disease, Sierra Leone. CSF, cerebrospinal fluid.
